# Nintedanib downregulates the transition of cultured systemic sclerosis fibrocytes into myofibroblasts and their pro-fibrotic activity

**DOI:** 10.1186/s13075-021-02555-2

**Published:** 2021-08-03

**Authors:** Maurizio Cutolo, Emanuele Gotelli, Paola Montagna, Samuele Tardito, Sabrina Paolino, Carmen Pizzorni, Alberto Sulli, Vanessa Smith, Stefano Soldano

**Affiliations:** 1 Laboratory of Experimental Rheumatology and Academic Division of Clinical Rheumatology, Department of Internal Medicine, University of Genova, IRCCS San Martino Polyclinic Hospital, Genoa, Italy; 2 Department of Rheumatology, Ghent University Hospital, Ghent, Belgium; 3 Department of Internal Medicine, Ghent University, Ghent, Belgium; 4 Unit for Molecular Immunology and Inflammation, VIB Inflammation Research Center (IRC), Ghent, Belgium

**Keywords:** Fibrocytes, Fibrosis, Tyrosine kinase inhibitor, Systemic sclerosis

## Abstract

**Background:**

Circulating fibrocytes are an important source of fibroblasts and myofibroblasts, which are involved in fibrotic processes, including systemic sclerosis (SSc).

The study aimed to investigate the effect of nintedanib (a tyrosine kinase inhibitor) in inhibiting the in vitro transition of circulating SSc fibrocytes into myofibroblasts and their pro-fibrotic activity.

**Methods:**

Circulating fibrocytes were obtained from 18 SSc patients and 5 healthy subjects (HSs). Cultured SSc fibrocytes were maintained in growth medium (untreated cells) or treated with nintedanib 0.1 and 1 μM for 3 and 24 h. Fibroblast-specific protein-1 (S100A4) and α-smooth muscle actin (αSMA), as markers of fibroblast/myofibroblast phenotype, together with type I collagen (COL1) and fibronectin (FN), were investigated by qRT-PCR and Western blotting. Non-parametric tests were used for statistical analysis.

**Results:**

Significantly elevated gene and protein expressions of αSMA, S100A4, COL1, and FN were observed in SSc fibrocytes compared to HS fibrocytes (gene: αSMA *p* < 0.001; others *p* < 0.0001; protein: all *p* < 0.05). Interestingly, an increased gene and protein expression of αSMA and S100A4 was found in fibrocytes from SSc patients positive for anti-Scl70 and with interstitial lung disease (ILD) (Scl70^+^ILD^+^) compared to Scl70^−^ILD^−^ patients (S100A4: gene: *p* < 0.01; protein: *p* < 0.05), whereas no differences were observed for COL1 and FN.

Nintedanib reduced gene and protein expression of αSMA, S100A4, COL1, and FN in SSc fibrocytes compared to untreated ones with different statistical significance.

Noteworthy, nintedanib significantly downregulated gene and protein expression of αSMA, S100A4, COL1, and FN in Scl70^+^ILD^+^ fibrocytes (all *p* < 0.05), whereas only that of S100A4 and FN was significantly downregulated (*p* < 0.05) in Scl70^−^ILD^−^ fibrocytes compared to the related untreated cells.

**Conclusions:**

Nintedanib seems to downregulate in vitro the transition of fibrocytes into myofibroblasts and their pro-fibrotic activity, particularly in cells isolated from Scl70^+^ILD^+^ SSc patients.

**Supplementary Information:**

The online version contains supplementary material available at 10.1186/s13075-021-02555-2.

## Introduction

Systemic sclerosis (SSc) is a complex chronic and progressive autoimmune connective tissue disease whose pathophysiology is characterized by microvascular damage, immune-inflammatory response, and diffuse fibrosis at the level of the skin and internal organs, including lung and heart [1]. Fibrosis is primarily determined by the persistent activation and transition of fibroblasts into α-smooth muscle actin (αSMA) positive myofibroblasts, which are considered the key effector cells of the excessive and progressive deposition of extracellular matrix (ECM) macromolecules, such as type I collagen (COL1) and fibronectin (FN) [2–4]. Inhibition of accumulation, activation, and transition of fibroblasts into myofibroblasts might contribute to reduce the fibrotic process and potentially to alter the natural development of many important fibrotic diseases, including SSc [5, 6]. Myofibroblasts may also differentiate from various other precursor cells, including epithelial cells, endothelial cells, pericytes, and fibrocytes [7, 8].

Fibrocytes are thought to be circulating fibroblast precursors expressing both hematopoietic and stromal cell markers (CD34, CD45, MHC class II), as well as COL1, which are used as the minimum criteria for identifying these cells in in vitro cultures, together with the surface expression of C-X-C chemokine receptor type 4 (CXCR4) [4, 9, 10].

Given the complexity of the fibrotic process in SSc, a single pathway intervention is unlikely to be curative, and polypharmacologic interventions are considered to be promising anti-fibrotic approaches despite the theoretically increased risk of adverse effects. The most relevant example to date is nintedanib [7].

Nintedanib is an oral, potent small-molecule tyrosine kinase inhibitor targeting platelet-derived growth factor receptor (PDGFR) A and B, fibroblast growth factor receptor (FGFR) 1-3, vascular endothelial growth factor receptor (VEGFR) 1-3, Src, Lyn, Lck, and colony-stimulating factor 1 receptor (CSF1R) [11]. By binding to the intracellular ATP binding site, nintedanib inhibits the activation of the intracellular signal transduction pathways mediated by these receptors [11]. In a mouse model of lung fibrosis, this tyrosine kinase inhibitor was observed to inhibit macrophage and fibroblast activation and to prevent fibrocyte migration [12–14]. Nintedanib was recently approved for the treatment of idiopathic pulmonary fibrosis (IPF) [15]. Moreover, the SENSCIS (Safety and Efficacy of Nintedanib in Systemic Sclerosis) trial showed that nintedanib has a beneficial effect in patients with interstitial lung disease (ILD) associated with SSc [15, 16].

Based on these observations, the aim of the study was to investigate in vitro the effect of nintedanib on the transition of cultured circulating fibrocytes, isolated from SSc patients, into pro-fibrotic myofibroblasts.

## Methods

### Isolation, in vitro culture and treatment of fibrocytes

Circulating fibrocytes were isolated from 18 SSc patients (16 females and 2 males, mean age 58 ± 11 years) who fulfilled the 2013 ACR/EULAR criteria for SSc diagnosis and from 5 voluntary age-matched healthy subjects (HSs, 4 females and 1 male). The SSc patients, undergoing complete disease staging in a day-hospital setting at the Rheumatology Division of Genoa University, including the nailfold videocapillaroscopy (NVC) pattern screening [1], were enrolled into the study after written informed consent and Ethical Committee approval (237REG2015, amendment number: 002-28/05/2018). The peripheral blood mononuclear cells (PBMCs) were isolated by density gradient centrifugation using Ficoll-Paque (Sigma-Aldrich, Milan, Italy) in accordance with the manufacturer’s protocol, and then plated on fibronectin-coated dishes in growth medium Dulbecco’s modified Eagle’s medium (DMEM) with 20% fetal bovine serum (FBS), 1% penicillin-streptomycin, and 1% L-glutamine (Euroclone, Milan, Italy). After overnight culture, non-adherent cells were removed and adherent fibrocytes were maintained in growth medium for 8 days (T8) [9, 17]. Growth medium was changed every 2 days. Cultured fibrocytes were preliminary characterized at T8 as CD45^+^COL1^+^CXCR4^+^ by Flow Cytometry, in accordance with our previous study as well as other studies [4, 9, 10, 17].

A part of T8-cultured SSc fibrocytes was treated for 3 and 24 h with nintedanib (Boehringer Ingelheim GmbH & Co. KG, Biberach, Germany) at the concentrations of 0.1 μM and 1 μM in growth medium, in accordance with recent in vitro studies [12, 14]. Another part of SSc fibrocytes was maintained in growth medium without any treatment and used as untreated cells. At the same time, T8-cultured fibrocytes isolated from HSs were maintained in growth medium without any treatment.

### Quantitative real-time polymerase chain reaction (qRT-PCR)

Total RNA was extracted with NucleoSpin RNA/protein (Macherey-Nagel, Duren, Germany) and quantified by NanoDrop (Thermo Scientific, Wilmington, USA), which also evaluates RNA integrity.

The qRT-PCR was performed on an Eppendorf Realplex 4 Mastercycler using the Real MasterMix SYBR Green detection system (Eppendorf, Milan, Italy) in a total volume of 10 μl loaded in triplicate. Primers for fibroblast and myofibroblast markers, fibroblast-specific protein-1 (S100A4, NM_002961) and αSMA (NM_001613), as well as COL1 (NM_000088), FN (NM_002026), CXCR4 (NM_00100854), and β-actin (NM_001101, housekeeping gene) were supplied by Primerdesign (Primerdesign, UK).

The melting curve was performed to confirm the specificity of the SYBR green assay. The qRT-PCR was performed on each independent in vitro experiment on cultures of fibrocytes isolated from all the enrolled SSc patients and HSs. Gene expression values were calculated using the comparative ΔΔCT method [18].

### Western blotting

Proteins were extracted using NucleoSpin RNA/protein (Macherey-Nagel) and quantified by the Bicinchoninic acid method. For each experimental condition, 30 μg of proteins were separated by electrophoresis on Bis-Tris gel (GenScript Biothec, Leiden, Netherlands) and transferred onto Hybond-C-nitrocellulose membranes (Life Technologies Ltd. Paisley, UK).

After 1 h in blocking solution (SuperBlock Blocking buffer in PBS, Thermo Scientific), membranes were incubated overnight at 4 °C with the following primary antibodies: anti-human αSMA (dilution 1:300, Sigma-Aldrich), S100A4 (dilution 1:100, Santa Cruz Biotechnology, Dallas, USA), COL1 (dilution 1:600, Proteintech), and FN (dilution 1:2,000, Sigma-Aldrich). The membranes were subsequently incubated with (HRP)-conjugated secondary antibodies (dilution 1:2,000; Cell Signaling, MA, USA). To confirm similar loading of protein samples into the gels and the efficiency in the electrophoretic transfer, membranes were incubated with primary HRP-conjugated antibody to human glyceraldehyde 3-phosphate dehydrogenase (GAPDH) (dilution 1:2,000; Santa Cruz Biotechnology). Protein synthesis was detected using the enhanced chemiluminescence system (Luminata Crescendo, Millipore), and the densitometric analysis was performed by the UVITEC Image Analysis System (UVITEC, Cambridge, UK).

### Statistical analysis

The statistical analysis was carried out by non-parametric Mann-Whitney test to compare gene and protein expression of the investigated molecules between cultured untreated fibrocytes isolated from HSs and SSc patients. Non-parametric Wilcoxon test was used to compare paired treatments in the in vitro experiments of cultured SSc fibrocytes treated with or without nintedanib. Any *p* value lower than 0.05 was considered as statistically significant. Results of qRT-PCR and Western blotting were analyzed and graphically reported as median with range.

## Results

Demographic and clinical parameters are reported in Tables 1 and 2. Mean disease duration ± standard deviation (SD) of enrolled SSc patients was 9.7 ± 6.9 years. The specific SSc antibody profile revealed ten patients with anti-topoisomerase I antibody positivity (Scl70^+^) whereas eight patients were Scl70^−^. The evaluation at lung high resolution CT scan revealed eleven patients characterized by ILD, whereas seven patients did not show ILD. All Scl70^+^ patients were characterized by ILD and six patients showed a disease duration longer than 13 years, whereas four patients showed a disease duration shorter than 10 years. Among the Scl70^−^ patients, seven patients did not show ILD and four of them were characterized by a disease duration shorter than 5 years, whereas three showed a disease duration longer than 7 years. Moreover, one Scl70^−^ patient was characterized by ILD and a disease duration shorter than 5 years.

**Table 1:** Summary of demographic and clinical parameters of SSc patients

Mean age ± standard deviation (years)	58 ± 11
Sex (F/M)	16/2
Mean Disease duration (years)	9.7 ± 6.9
Anti-Scl70 (*n* = %)	10 (55.5%)
CENP-A and CENP-B (*n* = %)	8 (44.5%)
ILD at CTscan (Yes/No)	11 (61.1%) / 7 (38.9%)
Skin involvement (lcSSc / dcSSc)	14 (77.7%) / 4 (22.3%)
NVC patterns (E/A/L)	2 (11.1%) / 10 (55.5%) / 6 (33.4%)
Therapy	No immunosuppressor = 7 (38.9%)
	MMF = 7 (38.9%)
	MTX = 2 (11.1%)
	Cyclosporine = 2 (11.1%)
	Endothelin receptor antagonists = 8 (44.4%)

Demographic and clinical parameters of enrolled SSc patients. Data include the age, female and male (F/M) ratio, skin involvement, nailfold videocapillaroscopy pattern of microan giopathy and auto-antibody profile. Data are expressed as means ± standard deviations (SD) or numbers (percentages of the total population). *lcSSc* “limited” cutaneous skin involve ment, *dcSSc* “diffuse” cutaneous skin involvement, *NVC* nailfold videocapillaroscopy, *E* “Early” *NVC* pattern of microangiopathy, *A* “Active” *NVC* pattern of microangiopathy, *L* “Late” *NVC* pattern of microangiopathy, *CENP* anti-centromere antibodies, *Scl70* anti-topoisomerase-I antibodies, *MMF* mycophenolate mofetil, *MTX* methotrexate

**Table 2:** Detailed clinical parameters of enrolled SSc patients

Sample ID	Sex (F/M)	Disease duration (years)	Auto-antibody profile	NVC pattern (E/A/L)	Skin involvement (lcSSc/dcSSc)	ILD (Yes/No)	GI involvement (Yes/No)	PAH (Yes/No)	Renal involvement (Yes/No)	DUs (Yes/No)	FVC	DLCO
SSc1	F	23	Scl70	L	dcSSc	Yes	Yes	Yes	Yes	Yes	77	44
SSc2	F	23	Scl70	L	lcSSc	Yes	Yes	Yes	No	Yes	/	/
SSc3	F	16	Scl70	A	lcSSc	Yes	Yes	Yes	No	Yes	88	93
SSc4	F	14	Scl70	A	lcSSc	Yes	No	No	Yes	No	97	53
SSc5	F	4	Scl70	L	dcSSc	Yes	No	No	No	Yes	108	70
SSc6	F	11	CENP-A/B	A	lcSSc	No	No	No	Yes	No	85	85
SSc7	F	3	CENP-A/B	A	lcSSc	No	No	No	Yes	No	110	97
SSc8	F	15	CENP-A/B	A	lcSSc	No	Yes	No	Yes	No	119	75
SSc9	M	13	Scl70	E	lcSSc	Yes	No	No	Yes	No	93	90
SSc10	F	2	CENP-A/B	A	lcSSc	Yes	Yes	No	Yes	No	113	91
SSc11	F	16	Scl70	L	dcSSc	Yes	Yes	No	No	Yes	26	27
SSc12	M	6	Scl70	A	lcSSc	Yes	Yes	No	No	Yes	74	92
SSc13	F	1	Scl70	L	lcSSc	Yes	Yes	No	No	Yes	68	47
SSc14	F	4	CENP-A/B	L	dcSSc	No	Yes	No	No	Yes	52	/
SSc15	F	4	CENP-A/B	A	lcSSc	No	No	No	No	No	119	142
SSc16	F	4	CENP-A/B	E	lcSSc	No	No	No	No	Yes	103	93
SSc17	F	7	CENP-A/B	A	lcSSc	No	Yes	No	Yes	No	93	102
SSc18	F	9	Scl70	A	lcSSc	Yes	Yes	Yes	Yes	Yes	86	47

Detailed clinical parameters of each enrolled SSc patient. Data include disease duration; autoantibody profile: anti-topoisomerase I (Scl70) antibodies, and anti-centromere antibodies polypeptides A and B (CENP-a and CENP-B); pattern of microangiopathy at nailfold videocapillaroscopy (NVC); “limited” (lcSSc) and “diffuse” (cdSSc) cutaneous skin involvement, gastrointestinal (GI) and renal involvements; presence of interstitial lung disease (ILD); pulmonary arterial hypertension (PAH); digital ulcers (DUs); forced vital capacity (FVC) and diffusing capacity of the lungs for carbon monoxide (DLCO)

The sign “/” indicates that patients was unable to complete the functional test

Considering the skin involvement, fourteen patients were characterized by a “limited” cutaneous skin involvement (lcSSc), whereas four patients showed a “diffuse” cutaneous skin involvement (dcSSc). The evaluation of the pattern of microvascular damage by NVC revealed two patients with a NVC patter “Early” (one Scl70^+^ and one Scl70^−^), ten patients with a NVC pattern “Active” (four Scl70^+^ and six Scl70^−^), and six patients with a NVC patter “Late” (five Scl70^+^ and one Scl70^−^). Concerning other organs, eleven patients showed a gastrointestinal involvement, nine patients were characterized by renal involvement and only four patients had pulmonary arterial hypertension (all Scl70+; two of them with NVC patter “Active” and two with NVC patter “Late”). Finally, ten patients were characterized by the presence of digital ulcers. Table 2 shows all detailed parameters of the enrolled SSc patients.

### Expression of fibroblast/myofibroblast phenotype markers and ECM macromolecules in fibrocytes isolated from SSc patients Scl70^+^ and with ILD.

**Fig. 1:**
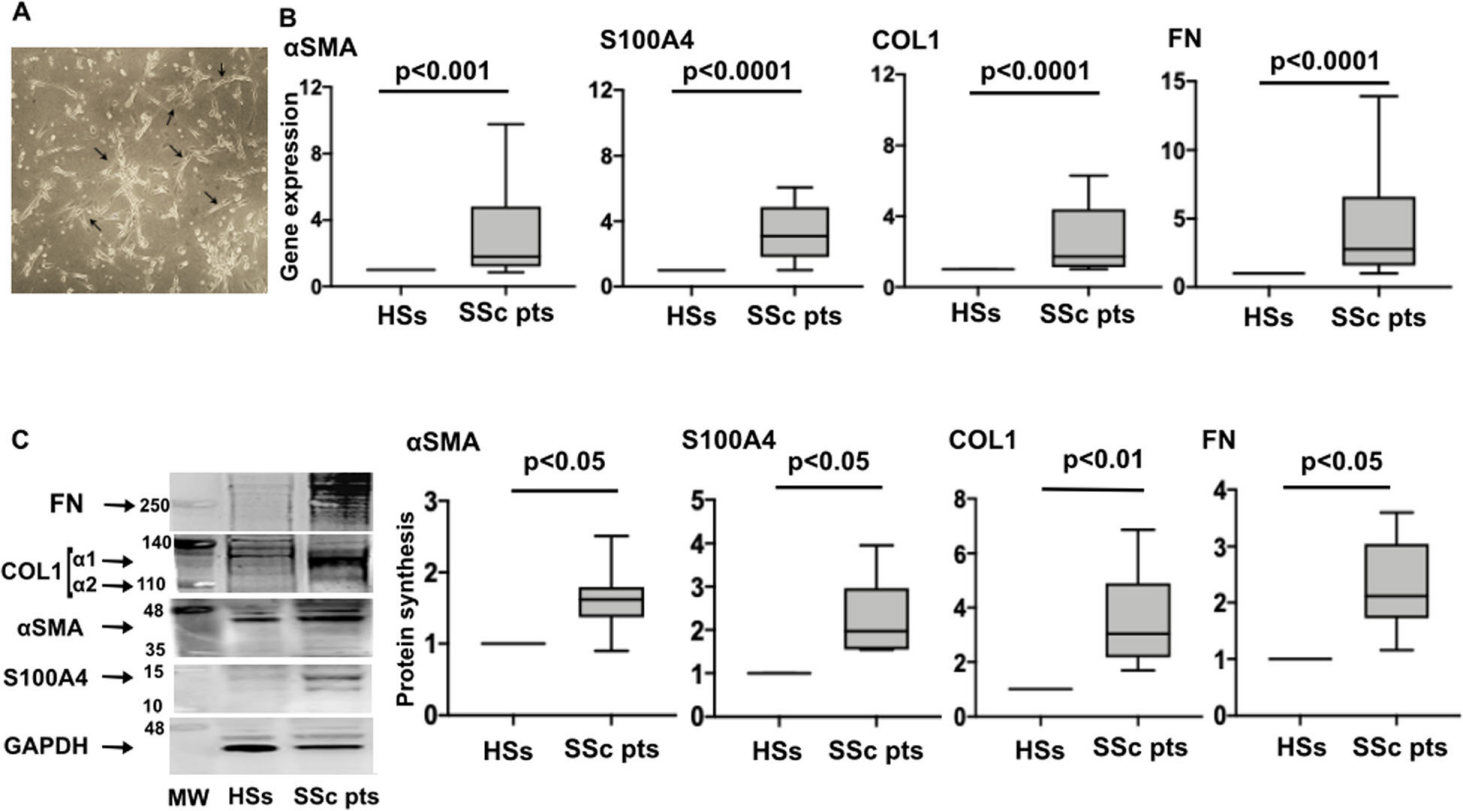
Gene and protein expression of myofibroblast phenotype markers and ECM macromolecules in SSc and HS fibrocytes. **A** Representative image of fibrocytes isolated from SSc patients evaluated by phase-contrast light microscopy at × 10 magnification. Fibrocytes (indicated by black arrows) appear as elongated cells characterized by a spindle-shaped morphology. **B** Quantitative real time polymerase chain reaction (qRT-PCR) of the gene expression of αSMA, S100A4, COL1, and FN in cultures of fibrocytes isolated from 18 SSc patients and 5 healthy subjects maintained in normal growth medium without any treatment. Gene expression corresponds to the expression level (fold-increase) of the target gene of SSc fibrocytes compared to that of HS fibrocytes, taken as the unit value by definition [18]. **C** Evaluation by Western blotting and related densitometric analysis of αSMA, S100A4 COL1, FN, and glyceraldehyde 3-phosphate dehydrogenase (GAPDH) in cultures of fibrocytes isolated from 12 SSc patients and 5 healthy subjects maintained in normal growth medium without any treatment. For each sample, the values of αSMA, S100A4, COL1, and FN are normalized to that of the corresponding GAPDH. The resulting value of each sample of SSc fibrocytes is compared to that isolated from HS fibrocytes (taken as the unit value). Data are reported as median with range

As shown by phase-contrast light microscopy analysis, after 8 days of culture in DMEM with 20% of FBS, adherent fibrocytes showed a spindle-shaped morphology (Fig. 1A).

The SSc fibrocytes were characterized by a significant higher gene expression and protein synthesis of αSMA, COL1 and FN compared to HS fibrocytes (gene: *p* < 0.001 for αSMA and *p* < 0.0001 for ECM macromolecules; protein: *p* < 0.01 for COL1 and *p* < 0.05 for all other molecules) (Fig. 1B, C). Moreover, SSc fibrocytes showed a significant higher gene and protein expression of S100A4 (*p* < 0.0001; *p* < 0.05 vs. HS fibrocytes, respectively), as specific fibroblast phenotype marker (Fig. 1B, C).

In accordance with the antibody profile and the presence or absence of ILD at CT scan, SSc patients were grouped as either Scl70^+^ patients with ILD (Scl70^+^ILD^+^) or Scl70^−^ patients without ILD (Scl70^−^ILD^−^).

**Fig. 2:**
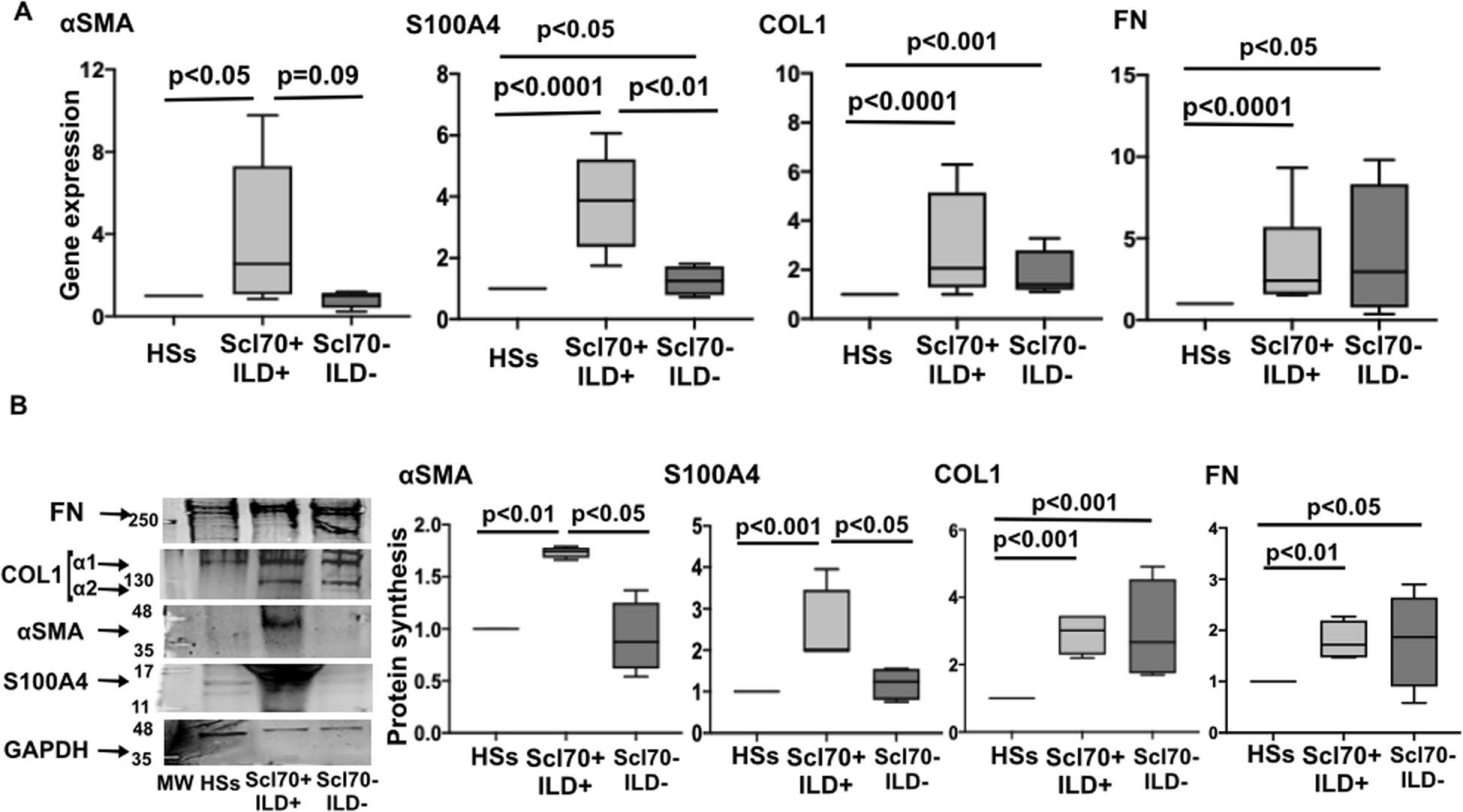
Gene and protein expression of myofibroblast phenotype markers and ECM macromolecules in Scl70^+^ILD^+^ and SSc70^−^ILD^−^ fibrocytes. **A** Quantitative real time polymerase chain reaction (qRT-PCR) of αSMA, S100A4, COL1, and FN in cultures of fibrocytes isolated from nine SSc patients with anti-Scl70 antibody positivity and interstitial lung disease (Scl70^+^ILD^+^), five patients negative for anti-Scl70 without ILD (Scl70^−^ILD^−^), and five healthy subjects (HSs) maintained in normal growth medium without any treatment. Gene expression corresponds to the expression level (fold-increase) of the target gene of Scl70^+^ILD^+^and Scl70^−^ILD^−^ fibrocytes compared with that of HS fibrocytes taken as the unit value by definition [18]. Data are expressed as median with range. **B** Evaluation by Western blotting and related densitometric analysis of αSMA, S100A4 COL1, FN, and glyceraldehyde 3-phosphate dehydrogenase (GAPDH) in cultures of fibrocytes isolated from nine Scl70^+^ILD^+^, five patients negative for anti-Scl70 without ILD (Scl70^−^ILD^−^), and five healthy subjects (HSs) maintained in normal growth medium without any treatment. For each sample, the values of αSMA, S100A4, COL1, and FN are normalized to that of the corresponding GAPDH. The resulting value of each sample of SSc fibrocytes is compared to that isolated from HS fibrocytes (taken as the unit value). Data are reported as median with range

Fibrocytes obtained from nine of the ten enrolled Scl70^+^ILD^+^ patients were characterized by a significant higher gene and protein expression of αSMA, S100A4, COL1, and FN compared to HS fibrocytes (gene: *p* < 0.05 for αSMA; *p* < 0.0001 for the other molecules; protein: *p* < 0.01 for αSMA and FN; *p* < 0.001 for S100A4 and COL1) (Fig. 2). At the same time, fibrocytes obtained from five of the seven Scl70^−^ILD^−^ patients showed a significantly increased gene expression of S100A4, COL1, and FN compared to HS fibrocytes (*p* < 0.05; *p* < 0.001; *p* < 0.05), whereas no difference in the αSMA gene expression was observed (Fig. 2A). Moreover, Scl70^−^ILD^−^ fibrocytes showed a significant increase in protein synthesis of COL1 and FN compared to HS fibrocytes (*p* < 0.001 for COL1, *p* < 0.05 for FN) (Fig. 2B). Of note, the gene expression of αSMA and S100A4 was higher in Scl70^+^ILD^+^ fibrocytes than in Scl70^−^ILD^−^ fibrocytes (*p* < 0.01 for S100A4) (Fig. 2A). Moreover, Scl70^+^ILD^+^ fibrocytes were characterized by a significantly higher protein synthesis of αSMA and S100A4 compared to Scl70^−^ILD^−^ fibrocytes (*p* < 0.05 for both molecules) (Fig. 2B). No statistically significant difference in COL1 and FN gene and protein expressions between Scl70^+^ILD^+^ and Scl70^−^ILD^−^ fibrocytes were observed (Fig. 2). The median (with range) of the gene expression levels is summarized in Supplementary Table IA.

### Effects of nintedanib on gene expression and protein synthesis of αSMA and ECM macromolecules in cultured SSc fibrocytes.

**Fig. 3:**
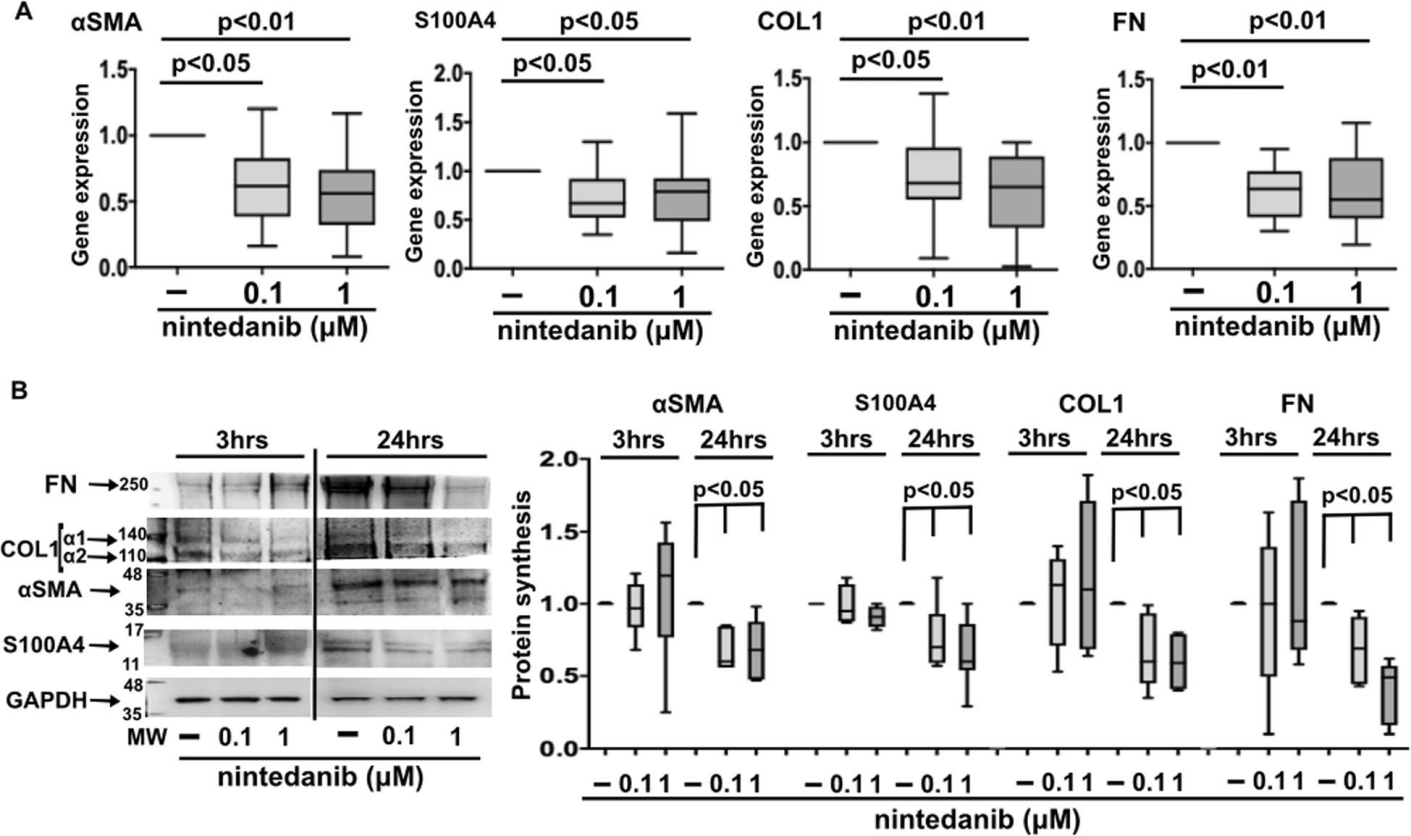
Gene and protein expression of myofibroblast phenotype markers and ECM macromolecules in nintedanib-treated SSc fibrocytes. **A** Quantitative real time polymerase chain reaction (qRT-PCR) of the gene expression of αSMA, S100A4, COL1, and FN in cultures of fibrocytes isolated from SSc patients maintained in normal growth medium without any treatment and treated with nintedanib at the concentrations of 0.1 μM and 1 μM for 3 h. The qRT-PCR is performed on 16 independent in vitro experiments derived from fibrocytes isolated from each enrolled SSc patient. Gene expression corresponds to the expression level (fold-increase) of the target gene of nintedanib-treated SSc fibrocytes compared with that of untreated cells, taken as the unit value by definition [18]. **B** Western blotting and related densitometric analysis of protein synthesis of αSMA, S100A4, COL1, FN, and GAPDH in SSc fibrocytes maintained in normal growth medium without any treatment and treated with nintedanib at the concentrations of 0.1 μM and 1 μM for 3 and 24 h. Western blotting is performed on 10 independent in vitro experiments with fibrocytes isolated from 6 SSc patients. For each experimental condition, the value for the synthesis of αSMA, S100A4, COL1, and FN is normalized to that of the corresponding GAPDH. The resulting value of each treatment is compared with that of the related untreated cells (taken as unit value). Data of qRT-PCR and Western blotting are expressed as median with range

In SSc fibrocytes, nintedanib 0.1 μM and 1 μM downregulated the gene expression of αSMA, S100A4, COL1, and FN after 3 h of treatment compared to untreated cells (*p* < 0.05 and *p* < 0.01 for αSMA; *p* < 0.05 for S100A4; *p* < 0.05 and *p* < 0.01 for COL1; *p* < 0.01 for FN) (Fig. 3A). The median (with range) of the gene expression levels is summarized in Supplementary Table IB.

At protein level, in SSc fibrocytes both concentrations of nintedanib significantly reduced the synthesis of αSMA, S100A4, COL1, and FN primarily after 24 h of treatment compared to untreated fibrocytes (*p* < 0.05 for all proteins for both concentrations) (Fig. 3B). No significant modulatory effect of nintedanib on the synthesis of all these proteins was observed in SSc fibrocytes treated for 3 h compared to untreated cells (Fig. 3B).

### Effects of nintedanib on the gene expression of αSMA and ECM macromolecules in cultured fibrocytes isolated from Scl70^+^ILD^+^patients.

**Fig. 4:**
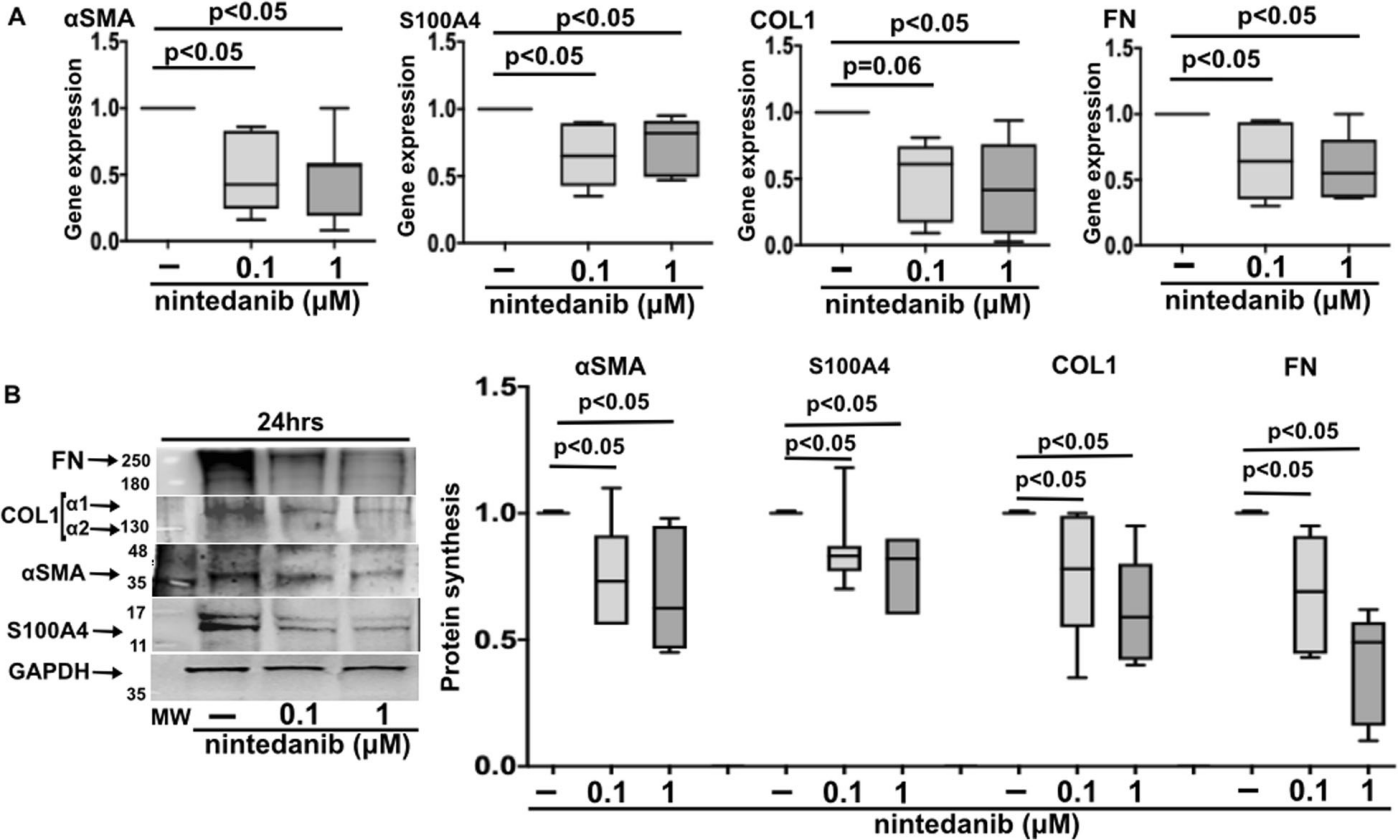
Gene and protein expression of myofibroblast phenotype markers and ECM macromolecules in Scl70^+^ILD^+^ fibrocytes treated with nintedanib. **A** Quantitative real time polymerase chain reaction (qRT-PCR) of αSMA, S100A4, COL1, and FN in cultures of fibrocytes isolated from patients with anti-Scl70 positivity and ILD (Scl70^+^ILD^+^) maintained in normal growth medium without any treatment and treated with nintedanib at the concentrations of 0.1 μM and 1 μM for 3 h. The qRT-PCR is performed on eight independent in vitro experiments derived from fibrocytes obtained from each enrolled Scl70^+^ILD^+^ patient. Gene expression corresponds to the expression level (fold-increase) of the target gene of nintedanib-treated SSc fibrocytes compared with that of untreated cells, taken as the unit value by definition [18]. Data are expressed as median with range. **B** Western blotting and related densitometric analysis of protein synthesis of αSMA, S100A4, COL1, FN, and GAPDH in cultured Scl70^+^ILD^+^ fibrocytes maintained in normal growth medium without any treatment and treated with nintedanib at the concentrations of 0.1 μM and 1 μM for 24 h. Western blotting is performed on eight independent in vitro experiments. For each experimental condition, the value for the synthesis of αSMA, S100A4, COL1, and FN is normalized to that of the corresponding GAPDH. The resulting value of each treatment is compared with that of the related untreated cells (taken as unit value)

In fibrocytes isolated from Scl70^+^ILD^+^ patients, nintedanib 0.1 μM and 1 μM significantly downregulated the gene expression of αSMA, S100A4, and FN compared to untreated cells after 3 h of treatment (*p* < 0.05 for both concentrations relative to all molecules) (Fig. 4A). Moreover, COL1 gene expression was also downregulated by nintedanib treatment, but significantly only at the higher concentration of 1 μM (*p* < 0.05 vs. untreated cells) (Fig. 4A). At protein level, both concentrations of nintedanib significantly reduced the synthesis of αSMA, S100A4, COL1, and FN after 24 h of treatment compared to untreated cells (*p* < 0.05 for both concentrations relative to all molecules) (Fig. 4B).

**Fig. 5:**
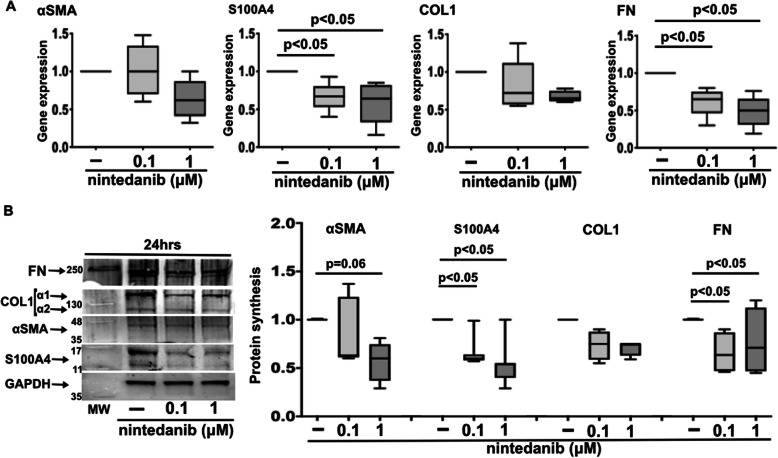
Gene and protein expression of myofibroblast phenotype markers and ECM macromolecules in Scl70^−^ILD^−^ fibrocytes treated with nintedanib. **A** Quantitative real time polymerase chain reaction (qRT-PCR) of αSMA, S100A4, COL1, and FN in cultures of fibrocytes isolated from patients negative for anti-Scl70 without ILD (Scl70^−^ILD^−^) maintained in normal growth medium without any treatment and treated with nintedanib at the concentrations of 0.1 μM and 1 μM for 3 h. The qRT-PCR is performed on five independent in vitro experiments derived from fibrocytes obtained from each enrolled Scl70^−^ILD^−^ patient. Gene expression corresponds to the expression level (fold-increase) of the target gene of nintedanib-treated SSc fibrocytes compared with that of untreated cells, taken as the unit value by definition [18]. Data are expressed as median with range. **B** Western blotting and related densitometric analysis of protein synthesis of αSMA, S100A4, COL1, FN, and GAPDH in cultured Scl70^−^ILD^−^ fibrocytes maintained in normal growth medium without any treatment and treated with nintedanib at the concentrations of 0.1 μM and 1 μM for 24 h. Western blotting is performed on five independent in vitro experiments. For each experimental condition, the value for the synthesis of αSMA, S100A4, COL1, and FN is normalized to that of the corresponding GAPDH. The resulting value of each treatment is compared with that of the related untreated cells (taken as unit value)

In fibrocytes isolated from Scl70^−^ILD^−^ patients, nintedanib 0.1 μM and 1 μM significantly downregulated both gene expression of S100A4 and FN after 3 h of treatment compared to untreated cells (*p* < 0.05 for both concentrations relative to all molecules) (Fig. 5A). In these fibrocytes, nintedanib downregulated (not significantly) the gene expression of αSMA and COL1, primarily at the higher tested concentration (Fig. 5A). The median (with range) of the gene expression levels is summarized in the Supplementary Table IB.

In Scl70^−^ILD^−^ fibrocytes, both concentrations of nintedanib reduced the protein synthesis of all the investigated molecules after 24 h of treatment, but significantly only for S100A4 and FN compared to untreated cells (*p* < 0.05 for both concentrations) (Fig. 5B).

Finally, in the fibrocytes isolated from SSc patients as well as from the two groups of Scl70^+^ILD^+^ and Scl70^−^ILD^−^ patients, nintedanib did not induced any modulatory effect on the gene and protein expression of CXCR4 (Supplementary Figure 1).

## Discussion

The results of the present in vitro study demonstrate that cultured SSc fibrocytes show a high pro-fibrotic activity compared to HS fibrocytes, as evidenced by their increased gene and protein expression of fibroblast/myofibroblast phenotype markers (S100A4 and αSMA), and ECM macromolecules (COL1 and FN), confirming that fibrocytes may serve as an important source of fibroblasts and myofibroblasts in SSc [17].

Nintedanib was found capable to downregulate the elevated gene and protein expression in cultured SSc fibrocytes.

The effect of nintedanib on the fibrocyte activity was investigated by Sato et al. in a previous study that demonstrated how this tyrosine kinase inhibitor blocked the fibrocyte differentiation from PBMCs, their migration induced by the growth factors PDGF, FGF2, and VEGF-A, and their ability to induce fibroblast differentiation [13].

In addition to these findings, also the results of our study highlight fibrocytes as an important target of the pharmacological activity of nintedanib in SSc patients. The attenuation of the transition of fibrocytes into myofibroblasts and of their overproduction of pro-fibrotic ECM molecules induced by nintedanib potentially contributes to ameliorate the fibrotic process that characterizes SSc, at least in vitro.

Another important result of our in vitro study is the significant over-expression and over-production of fibroblast/myofibroblast phenotype markers (S100A4 and αSMA) that seems more evident in fibrocytes isolated from Scl70^+^ILD^+^ patients compared to fibrocytes isolated from Scl70^−^ILD^−^ patients, as well as from HSs.

Of note, in these Scl70^+^ILD^+^ fibrocytes, nintedanib induced a significant downregulation of gene expression and protein synthesis of both fibroblast/myofibroblast markers as well as that of COL1 and FN; the downregulation was already evident at the lowest tested concentration of the tyrosine kinase inhibitor.

However, the downregulatory effect of nintedanib on the gene expression and protein synthesis of fibroblast/myofibroblast phenotype markers and ECM proteins was also observed in fibrocytes isolated from Scl70^−^ILD^−^ patients, where it significantly reduced S100A4 and FN, whereas the downregulation of αSMA and COL1 was not statistically significant, probably due to the small number of patients in this subgroup.

As known, in SSc patients, the presence of Scl70 is more frequently associated with pulmonary fibrosis (PF) and ILD or pulmonary arterial hypertension [19, 20].

In a study of Nihtyanova SI et al., it was demonstrated that the positivity for anti-Scl70 antibodies is predictive of the development of PF and these patients showed a higher hazard of death compared to patients positive for anti-centromere and anti-RNA polymerase antibodies [21].

Moreover, a more recent study by the same author demonstrated a time-dependent effect of anti-Scl70 antibodies on the hazard of clinically significant PF [22].

Some other studies have also suggested that in Scl70^+^ SSc patients, PF represents an earlier complication compared to patients with anti-RNA polymerase antibodies, who tend to develop PF later in the disease course [23, 24].

In the Nihtyanova’s study, the strongest positive association emerged between cardiac involvement and the anti-Scl70 antibody positivity beyond that with PF; moreover, within the Scl70^+^ patient group, those with dcSSc had a worse prognosis compared to lcSSc patients [25]. On the contrary, patients with an anticentromere antibody positivity and lcSSc had the best 20-year survival and a better long-term prognosis in terms of severity of internal organ involvement [25]. In our cohort of SSc patients, only three Scl70^+^patients were characterized by a dcSSc, making any statistical evaluation impossible, which represents a limitation in this study.

However, the progressive systemic fibrosis in ILD positive patients was also confirmed by the concomitant presence of the most advanced “Late” NVC pattern of microvascular damage that characterizes SSc patients with intensive fibrosis [26]. Based on these clinical considerations, the downregulatory effect of nintedanib on the transition of fibrocytes isolated from Scl70^+^ILD^+^ patients into myofibroblasts and their ECM overproduction might be a further supporting element for the beneficial effects exerted by nintedanib in reducing the progression of the ILD in SSc [15, 16, 27, 28]. However, the positivity for anti-Scl70 antibodies in some SSc patients may appear before or after the lung involvement, suggesting how this involvement might be associated also with other risk factors and pro-fibrotic mediators, such as other functional autoantibodies (anti-endothelin receptors, anti-angiotensin-II receptor, anti-endothelial cells, or anti-fibroblast antibodies) [29, 30].

In the evaluation of the transition process of those cells considered to be a source of fibroblasts, such as endothelial/epithelial cells and circulating fibrocytes, S100A4 is a recommended marker of fibroblast phenotype [31–33].

Recently, S100A4 was demonstrated to be expressed by αSMA^+^ cells in the lung interstitium of patients affected by IPF, playing a role in the development of this condition [34]. The results of the present in vitro study highlight how SSc fibrocytes seem to be characterized by a higher gene and protein expression of S100A4 compared to HS fibrocytes and further suggest how these cells might be already activated to undergo transition into fibroblasts [17].

The chemokine CXCR4 is one of the markers used to characterize fibrocytes, since it is expressed on almost all circulating fibrocytes and fibroblasts [35, 36]. Moreover, this chemokine is implicated in the migration of fibrocytes and fibroblasts allowing their recruitment to damaged tissues through the CXCL12/CXCR4 pathway [37, 38]. In accordance with this observation, the results of our in vitro study confirmed that CXCR4 is expressed on cultured SSc fibrocytes; however, the treatment with nintedanib did not modulate its gene expression and protein synthesis, suggesting that this tyrosine kinase inhibitor might not block the capability of fibrocytes to migrate into the tissue at least by interfering with the CXCL12/CXCR4 pathway.

## Conclusions

In conclusion, the results of this in vitro study show that nintedanib attenuates the activation and transition of cultured SSc fibrocytes into pro-fibrotic myofibroblasts. In addition, nintedanib reduces the fibroblast pro-fibrotic activity, particularly in those cells isolated from Scl70^+^ILD^+^ patients. A clinical exploration in such SSc patient subset it is warranted to confirm these promising in vitro results and to further justify the peculiar anti-fibrotic activity of nintedanib.

## Supplementary Information

**Additional file 1: Supplementary Table 1.** Gene expression values of fibroblast/myofibroblast phenotype markers and ECM macromolecules in cultured fibrocytes. (A) Expression levels of αSMA, S100A4, COL1, FN and CXCR4 in cultures of fibrocytes isolated from healthy subjects (HS), SSc patients, anti-Scl70^+^ patients with ILD (Scl70^+^ILD^+^) and anti-Scl70^−^ patients without ILD (Scl70^−^ILD^−^) by quantitative real time polymerase chain reaction (qRT-PCR). Gene expression corresponds to the expression level (fold-increase) of the target gene of SSc fibrocytes compared with that of HS fibrocytes, taken as the unit value by definition [18]. Data are expressed as median with range. (B) Expression levels of αSMA, S100A4, COL1, FN and CXCR4 in cultures of fibrocytes isolated from all SSc patients, Scl70^+^ILD^+^ patients and Scl70^−^ILD^−^ patients by quantitative real time polymerase chain reaction (qRT-PCR). Fibrocytes were maintained in normal growth medium without any treatment and treated with nintedanib at the concentrations of 0.1 μM and 1 μM for 3 h. Gene expression corresponds to the expression level (fold-increase) of the target gene of nintedanib-treated SSc fibrocytes compared with that of untreated cells, taken as the unit value by definition [18]. Data are expressed as median with range.

**Additional file 2: Supplementary Figure 1.** Gene and protein expression of CXCR4 in SSc fibrocytes after treatment with nintedanib. Gene expression of CXCR4 in cultures of fibrocytes maintained in normal growth medium without any treatment and treated with nintedanib at the concentrations of 0.1 μM and 1 μM for 3 h. (A) Fibrocytes isolated from SSc patients, (B) fibrocytes isolated from Scl70^+^ILD^+^ patients and (C) fibrocytes isolated from Scl70^−^ILD^−^ patients. Gene expression corresponds to the expression level (fold-increase) of the target gene of nintedanib-treated SSc fibrocytes compared with that of untreated cells, taken as the unit value by definition [18]. Data are expressed as median with range. (D) Western blotting and related densitometric analysis of protein synthesis of CXCR4 and GAPDH in cultures of fibrocytes obtained from SSc patients, Scl70^+^ILD^+^ patients and Scl70^−^ILD^−^ patients maintained in normal growth medium without any treatment and treated with nintedanib at the concentrations of 0.1 μM and 1 μM for 24 h. For each experimental condition, the value for the synthesis of CXCR4 is normalized to that of the corresponding GAPDH. The resulting value of each treatment is compared with that of the related untreated cells (taken as unit value).

## Data Availability

All data generated or analyzed during this study are included in this published article [and its supplementary information files].

## Abbreviations

SSc: Systemic sclerosis
αSMA: Alpha smooth muscle actin
ECM: Extracellular matrix
COL1: Type I collagen
FN: Fibronectin
CD34: Cluster of differentiation 34 and member of a family of single-pass transmembrane sialomucin proteins
CD45: Cluster of differentiation 45 or protein tyrosine phosphatase receptor type C
MHC: Major histocompatibility complex
CXCR4: CXC chemokine receptor type 4
PDGFR: Platelet derived growth factor receptor
FGFR: Fibroblast growth factor receptor
VEGFR: Vascular endothelial growth factor receptor
Src: Proto-oncogene tyrosine-protein kinase Src
Lyn: Tyrosine-protein kinase Lyn
Lck: Lymphocyte-specific protein tyrosine kinase
CSF1R: Colony-stimulating factor 1 receptor
ATP: Adenosine triphosphate
IPF: Idiopathic pulmonary fibrosis
SENSCIS: Safety and Efficacy of Nintedanib in Systemic Sclerosis
ILD: Interstitial lung disease
ACR: American College of Rheumatology
EULAR: European League Against Rheumatism
HSs: Healthy subjects
PBMCs: Peripheral blood mononuclear cells
DMEM: Dulbecco’s modified Eagle’s medium
FBS: Fetal bovine serum
μM: Micromolar
RNA: Ribonucleic acid
cDNA: Complementary deoxyribonucleic acid
qRT-PCR: Quantitative real time polymerase chain reaction
S100A4: S100 calcium-binding protein A4 or metastasin or fibroblast-specific protein-1
SYBR: Syber
ΔΔCt: Delta delta cycle threshold
HRP: Horseradish peroxidase
GAPDH: Glyceraldehyde 3- phosphate dehydrogenase
SD: Standard deviation
Scl70: Topoisomerase I antibody
CT: Computer tomography
lcSSc: Limited cutaneous skin involvement
dcSSc: Diffuse cutaneous skin involvement
NVC: Nailfold capillaroscopy
PDGF: Platelet-derived growth factor
FGF2: Fibroblast growth factor 2
VEGF-A: Vascular endothelial growth factor A
PF: Pulmonary fibrosis
CXCL12: Stromal cell derived factor 1 or chemokine (C-X-C motif) ligand 12
