# Correction: Nintedanib downregulates the transition of cultured systemic sclerosis fibrocytes into myofibroblasts and their pro-fibrotic activity

**DOI:** 10.1186/s13075-023-03117-4

**Published:** 2023-07-29

**Authors:** Maurizio Cutolo, Emanuele Gotelli, Paola Montagna, Samuele Tardito, Sabrina Paolino, Carmen Pizzorni, Alberto Sulli, Vanessa Smith, Stefano Soldano

**Affiliations:** 1 Laboratory of Experimental Rheumatology and Academic Division of Clinical Rheumatology, Department of Internal Medicine, University of Genova, IRCCS San Martino Polyclinic Hospital, Genoa, Italy; 2 Department of Rheumatology, Ghent University Hospital, Ghent, Belgium; 3 Department of Internal Medicine, Ghent University, Ghent, Belgium; 4 Unit for Molecular Immunology and Inflammation, VIB Inflammation Research Center (IRC), Ghent, Belgium

**Correction: Arthritis Res Ther 23, 205 (2021)**

**https://doi.org/10.1186/s13075-021-02555-2**

Following publication in Arthritis Research & Therapy of the original article entitled “Nintedanib downregulates the transition of cultured systemic sclerosis fibrocytes into myofibroblasts and their pro-fibrotic activity.” by Cutolo M et al., the authors need to include the statement of the co-author Professor Vanessa Smith in the Acknowledgements section of the revised manuscript in accordance with the Author guidelines of the journal as follows:

“Vanessa Smith is senior clinical investigator of the Research Foundation—Flanders (Belgium) (FWO) [1.8.029.20N].”

The original article [1] has been updated.

## References

[1] Cutolo M, Gotelli E, Montagna P (2021). Nintedanib downregulates the transition of cultured systemic sclerosis fibrocytes into myofibroblasts and their pro-fibrotic activity. Arthritis Res Ther.

