# Supplementary material for: Nintedanib downregulates the transition of cultured systemic sclerosis fibrocytes into myofibroblasts and their pro-fibrotic activity
Source: Arthritis Res Ther. 2021 Aug 3;23:205. doi: 10.1186/s13075-021-02555-2 (PMC8330043; doi:10.1186/s13075-021-02555-2)
Supplement: Supplementary file 1 — Additional file 1: Supplementary Table 1. Gene expression values of fibroblast/myofibroblast phenotype markers and ECM macromolecules in cultured fibrocytes. (A) Expression levels of αSMA, S100A4, COL1, FN and CXCR4 in cultures of fibrocytes isolated from healthy subjects (HS), SSc patients, anti-Scl70+ patients with ILD (Scl70+ILD+) and anti-Scl70− patients without ILD (Scl70−ILD−) by quantitative real time polymerase chain reaction (qRT-PCR). Gene expression corresponds to the expression level (fold-increase) of the target gene of SSc fibrocytes compared with that of HS fibrocytes, taken as the unit value by definition [18]. Data are expressed as median with range. (B) Expression levels of αSMA, S100A4, COL1, FN and CXCR4 in cultures of fibrocytes isolated from all SSc patients, Scl70+ILD+ patients and Scl70−ILD− patients by quantitative real time polymerase chain reaction (qRT-PCR). Fibrocytes were maintained in normal growth medium without any treatment and treated with nintedanib at the concentrations of 0.1 μM and 1 μM for 3 h. Gene expression corresponds to the expression level (fold-increase) of the target gene of nintedanib-treated SSc fibrocytes compared with that of untreated cells, taken as the unit value by definition [18]. Data are expressed as median with range. [file 13075_2021_2555_MOESM1_ESM.pdf]

A

	Cultured SSc fibrocytes vs. cultured HS fibrocytes	p-value	Cultured fibrocytes from $Scl70^{+}ILD^{+}$pts vs. cultured HS fibrocytes	p-value	Cultured fibrocytes from $Scl70^{-}ILD^{-}$pts vs. cultured HS fibrocytes	p-value	p-value cultured fibrocytes $Scl70^{+}ILD^{+}$pts vs. $Scl70^{-}ILD^{-}$pts
**αSMA**	**1.8** (1.2-4.83) vs. 1	**p=0.0008**	**2.56** (1.0-9.78) vs. 1	**p=0.04**	**1** (0.23-1.2) vs. 1	p>0.99	p=0.09
**S100A4**	**3.1** (1.8-6.1) vs. 1	**p<0.0001**	**3.87** (1.75-6.1) vs. 1	**p<0.0001**	**1.25** (0.72-1.81) vs. 1	**p=0.017**	**p=0.006**
**COL1**	**1.72** (1.0-6.3) vs. 1	**p<0.0001**	**2.07** (1.28-6.3) vs. 1	**p<0.0001**	**1.4** (1.11-3.28) vs. 1	**p=0.0003**	p=0.34
**FN**	**2.76** (1.57-13.9) vs. 1	**p<0.0001**	**2.76** (1.53-9.33) vs 1	**p<0.0001**	**2.95** (0.35-9.8) vs. 1	**p=0.017**	p=0.93
**CXCR4**	**0.81** (0.26-3.12) vs. 1	p=0.13	**0.72** (0.26-3.12) vs. 1	p=0.19	**0.91** (0.26-1.27) vs. 1	p=0.64	p>0.99

B

	Cultured SSc fibrocytes				
	Nintedanib 0.1μM vs. untreated	p value	Nintedanib 1μM vs. untreated	p value	p-value Nintedanib 0.1μM vs. Nintedanib 1μM
**αSMA**	**0.61** (0.16-0.82) vs. 1	**p=0.012**	**0.56** (0.08-0.74) vs. 1	**p=0.004**	p=0.48
**S100A4**	**0.67** (0.35-1.3) vs. 1	**p=0.016**	**0.79** (0.16-1.59) vs.1	**p=0.016**	p=0.9
**COL1**	**0.68** (0.09-1.38) vs. 1	**p=0.024**	**0.65** (0.02-1.0) vs. 1	**p=0.002**	p=0.3
**FN**	**0.63** (0.3-0.95) vs. 1	**p=0.0005**	**0.55** (0.19-1.16) vs. 1	**p=0.0024**	p=0.83
**CXCR4**	**0.90** (0.3-1.6) vs. 1	p=0.46	**1.06** (0.22-2.05) vs.1	p=0.37	p=0.21

	Cultured fibrocytes from $Scl70^{+}ILD^{+}$pts				
	Nintedanib 0.1μM vs. untreated	p value	Nintedanib 1μM vs. untreated	p value	p-value Nintedanib 0.1μM vs. Nintedanib 1μM
**αSMA**	**0.42** (0.16-0.86) vs. 1	**p=0.031**	**0.57** (0.08-1.0) vs. 1	**p=0.031**	p=0.81
**S100A4**	**0.65** (0.35-0.9) vs. 1	**p=0.037**	**0.82** (0.47-0.95) vs. 1	**p=0.031**	p=0.69
**COL1**	**0.61** (0.09-0.81) vs. 1	p=0.06	**0.41** (0.02-0.94) vs. 1	**p=0.031**	p=0.81
**FN**	**0.64** (0.3-0.9) vs. 1	**p=0.016**	**0.55** (0.36-1.0) vs. 1	**p=0.031**	p=0.47
**CXCR4**	**1.01** (0.62-1.27) vs.1	p>0.999	**0.9** (0.71-1.62) vs. 1	p=0.69	p=0.31

	Cultured fibrocytes from $Scl70^{-}ILD^{-}$pts				
	Nintedanib 0.1μM vs. untreated	p value	Nintedanib 1μM vs. untreated	p value	p-value Nintedanib 0.1μM vs. Nintedanib 1μM
**αSMA**	**1** (0.6-1.48) vs. 1	p>0.999	**0.62** (0.32-1.0) vs. 1	p=0.13	p=0.12
**S100A4**	**0.67** (0.4-0.93) vs. 1	**p=0.04**	**0.64** (0.16-0.85) vs. 1	**p=0.04**	p>0.99
**COL1**	**0.72** (0.55-1.38) vs. 1	p=0.31	**0.65** (0.6-0.78) vs. 1	p=0.16	p=0.63
**FN**	**0.65** (0.3-0.8) vs. 1	**p=0.016**	**0.5** (0.19-0.76) vs. 1	**p=0.016**	p=0.06
**CXCR4**	**0.82** (0.51-1.6) vs.1	p=0.87	**1.3** (0.9-2.05) vs.1	p=0.25	p=0.62
