# Supplementary figures and images for: Nintedanib downregulates the transition of cultured systemic sclerosis fibrocytes into myofibroblasts and their pro-fibrotic activity
Source: Arthritis Res Ther. 2021 Aug 3;23:205. doi: 10.1186/s13075-021-02555-2 (PMC8330043; doi:10.1186/s13075-021-02555-2)

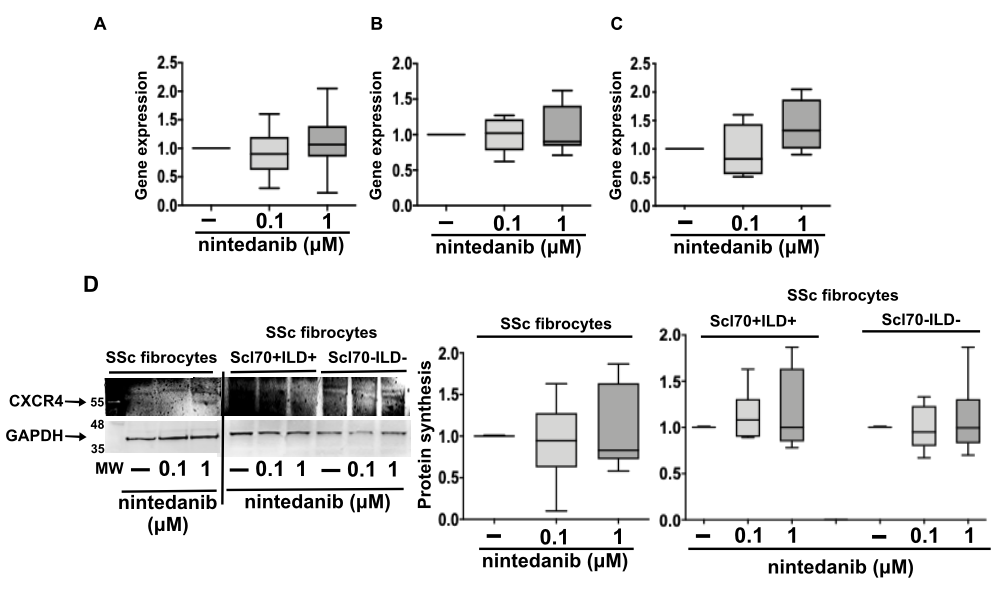

Supplement: Supplementary file 2 — Additional file 2: Supplementary Figure 1. Gene and protein expression of CXCR4 in SSc fibrocytes after treatment with nintedanib. Gene expression of CXCR4 in cultures of fibrocytes maintained in normal growth medium without any treatment and treated with nintedanib at the concentrations of 0.1 μM and 1 μM for 3 h. (A) Fibrocytes isolated from SSc patients, (B) fibrocytes isolated from Scl70+ILD+ patients and (C) fibrocytes isolated from Scl70−ILD− patients. Gene expression corresponds to the expression level (fold-increase) of the target gene of nintedanib-treated SSc fibrocytes compared with that of untreated cells, taken as the unit value by definition [18]. Data are expressed as median with range. (D) Western blotting and related densitometric analysis of protein synthesis of CXCR4 and GAPDH in cultures of fibrocytes obtained from SSc patients, Scl70+ILD+ patients and Scl70−ILD− patients maintained in normal growth medium without any treatment and treated with nintedanib at the concentrations of 0.1 μM and 1 μM for 24 h. For each experimental condition, the value for the synthesis of CXCR4 is normalized to that of the corresponding GAPDH. The resulting value of each treatment is compared with that of the related untreated cells (taken as unit value). [file 13075_2021_2555_MOESM2_ESM.tiff]
